# A decision aid to assist decisions on disclosure of mental health status to an employer: protocol for the CORAL exploratory randomised controlled trial

**DOI:** 10.1186/1471-244X-12-133

**Published:** 2012-08-31

**Authors:** Claire Henderson, Elaine Brohan, Sarah Clement, Paul Williams, Francesca Lassman, Oliver Schauman, Joanna Murray, Caroline Murphy, Mike Slade, Graham Thornicroft

**Affiliations:** 1 Health Service and Population Research Department P029, David Goldberg Centre, King's College London Institute of Psychiatry, De Crespigny Park, London, SE5 8AF, UK; 2 Department of Biostatistics, King's Clinical Trial Unit, King's College London, London, UK

**Keywords:** Decision aid, Disclosure of illness, Employment, Single blind, Randomised controlled trial

## Abstract

**Background:**

The UK Equality Act 2010 makes it unlawful for employers to ask health questions before making an offer of employment except in certain circumstances. While the majority of employers would prefer applicants to disclose a mental illness at the application stage, many people either wait until they have accepted the job and then disclose to an occupational health professional, or do not do so at all due to the anticipation of discrimination or a wish for privacy. However, non disclosure precludes the ability to request reasonable adjustments in the workplace or to make a claim of direct discrimination. Disclosure to employers is therefore a difficult decision. A recent pilot study by our group of the CORAL decision aid showed that it helped mental health service users clarify their needs and values regarding disclosure and led to reduction in decisional conflict. The present proof of concept trial aims to determine whether a full scale randomised controlled trial (RCT) is justifiable and feasible, and to optimise its design.

**Methods:**

In this single blind exploratory RCT in London, a total of 80 participants (inclusion criteria: age ≥18 years, on the caseload of a specialist employment adviser working with people with mental illness; referred to the adviser either from primary care via Improving Access to Psychological Therapies or secondary mental health service; currently seeking or interested in either paid or voluntary employment, and a Decisional Conflict Scale score of 37.5 or greater and stage of decision score 1–5) will be recruited from vocational advice services. After completing a baseline assessment, participants will be randomly assigned to one of two conditions (1) Use of the CORAL Decision Aid (DA) in addition to treatment as usual or (2) Treatment as usual. Those allocated to the DA condition will be given it to read and complete, and the researcher will be present to record the time taken and any content that causes confusion. Intervention participants may keep the decision aid but are discouraged from showing it to other service users to avoid contamination. Follow up interviews will be conducted at 3 months. Primary outcomes are: (i) stage of decision making score; (ii) decisional conflict scores and (iii) employment related outcomes. Secondary analyses will identify predictors of disclosure and qualitative analysis will explore the impact of the intervention.

**Discussion:**

A reduction in decisional conflict regarding disclosure leading to more effective job seeking activity could have significant economic consequences for people with mental illness in terms of employment rates and productivity.

**Trial registration number:**

NCT01379014 (ClinicalTrials.gov Identifier)

## Background

People with mental health problems frequently report discrimination in employment. In a US survey 61% (n = 1,301) felt they had been turned down for a job for which they are qualified when they disclosed their illness [1]. In the UK, 56% (n = 411) believed that they had definitely or possibly been turned down for a job in the past because of their mental health problems [2]. Similarly, in a recent international survey, 64% of participants (n = 736) reported stopping themselves from applying for work, training or education due to anticipated discrimination as a result of their diagnosis of schizophrenia [3]. Anticipated discrimination by employers also forms a barrier to seeking mental health care; this obstacle was reported more frequently than any other in a recent study [4].

Given these high frequencies of experienced and anticipated discrimination among people with mental health problems, many choose not to disclose their condition prior to being offered a job. This contrasts with employers’ reported preferences that they do so, for example 77% in a UK sample surveyed in 2009 [5]. However, with the introduction of the Equality Act 2010 it is now unlawful for employers in Britain to enquire about an applicant’s disability or health, until that person has either been offered a job or been included in a pool of candidates to be offered a job when a suitable position arises. Nothing in the Act prevents employers asking health related questions once recruitment decisions have been taken. This restriction is qualified by several exclusions and is only enforceable by the Equality and Human Rights Commission. However, its breach constitutes evidence of disability discrimination.

In some circumstances disclosure may be obligatory. For example, if a job environment is such that one’s disability could present a risk to one’s health and safety or that of colleagues [6]. However, in most cases the Act does not obligate disclosure of a mental illness prior to employment, so it is up to the job candidate to decide whether to do so. There are two important legal considerations [7]. First, a claim for direct discrimination or discrimination arising from disability can only be made where the employer knew or ought to have known that the person was disabled. Second, no duty arises to provide ‘reasonable adjustments’ if the employer does not know or could not reasonably be expected to know that a person has a disability. There are also many other considerations. Will colleagues misinterpret symptoms of mental illness [8] e.g. as substance misuse if one does not disclose? Will they gossip [9], ignore them [8] or interpret every expression of emotion as a symptom if they do? How much should one say, when and to whom? Disclosure of mental illness in the employment context is therefore a personal, multilayered and potentially difficult decision.

A decision aid (DA) is an educational intervention designed to help an individual make a specific and deliberate choice between two or more options. They are commonly used in medical decision-making when individuals need to choose between treatment or screening options [10]. A systematic review of 17 randomised trials of DAs found that, compared with controls, DAs produced higher knowledge, more active participation in decision making and lower levels of decisional conflict [11,12].

This suggests that a DA may be a useful intervention to increase knowledge about disclosure, reduce decisional conflict or increase active participation in this employment decision making.

We have recently developed a DA to assist people with mental health problems consider disclosure in the employment context (Conceal Or ReveAL, or CORAL). In a non-randomised pilot study [13], the CORAL DA demonstrated preliminary evidence of acceptability, feasibility and effectiveness in a group of 15 service users. We now wish to determine through a randomised controlled trial whether using the CORAL DA changes the user’s behaviour with respect to seeking or retaining employment in ways that result in higher levels of employment and greater use of workplace accommodations. These outcomes require larger sample sizes and a longer follow up period than are justifiable at this point. To determine whether such a trial is justifiable and feasible and to optimise its design, our next step is to conduct a proof of concept RCT.

## Methods

### Design

The CORAL trial is an individual-level single-blind RCT of the CORAL decision aid compared with a treatment as usual control for people receiving primary (Improving Access to Psychological Therapies (IAPT)) or secondary mental health care services in south east London. The total duration of the study will be two years, to allow for: regulatory approvals, recruitment; provision of the intervention; follow-up assessments; qualitative exploration of use of the DA; and data analysis, using intention to treat methods. Recruitment to the trial began in July 2011 and is due for completion in June 2012. Follow-up assessments began in October 2011 and will be completed by September 2012.

### Setting

The settings are in London at locations where vocational advisers employed or contracted by an NHS Foundation Trust work with clients from the Trust and from IAPT.

### Participants

**Figure 1:**
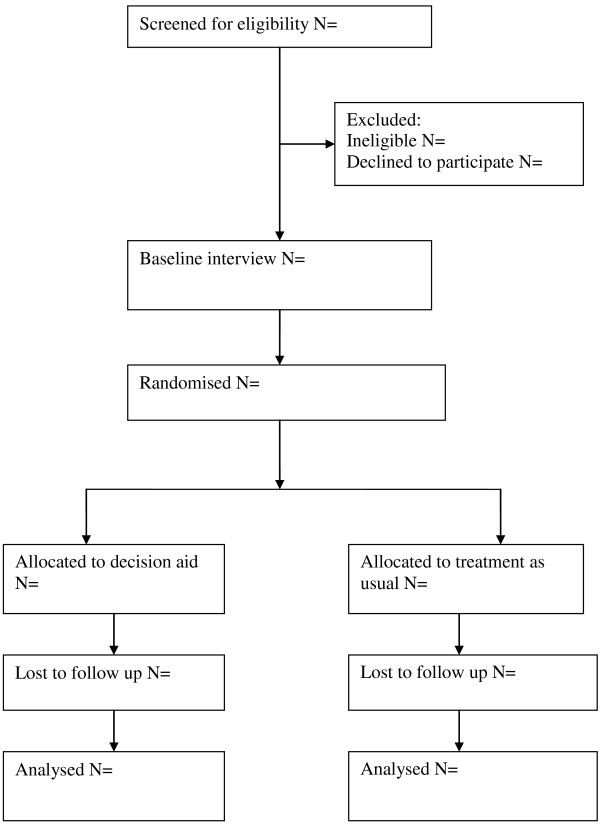
Consort flow chart of CORAL trial design.

Eligible service users will (i) be on the caseload of a specialist employment adviser working with people with mental illness; (ii) have been referred to the adviser either from the primary care based mental health service Improving Access to Psychological Therapies (IAPT) or secondary mental health care service; (iii) be aged 18 years or older; (iv) be currently seeking either paid or voluntary employment or interested in seeking paid or voluntary employment (v) have a Decisional Conflict Scale score of 37.5 or greater (showing at least moderate decisional conflict) and a Stage of Decision Scale score of 1–5 (showing the decision has not yet been reached); and (vi) give written, informed consent. We will exclude service users who have insufficient literacy in English to use the DA or who lack capacity to provide informed consent. Figure 1 shows the CONSORT flow chart for participants.

### Intervention

A researcher will meet with each participant in the intervention group and give them the DA to read and complete.

The DA has been designed for use independent from or as an adjunct to a clinical encounter [14]. It includes six sections [1]: the pros and cons of disclosure [2]; personal disclosure needs [3]. personal disclosure values [4]; when to tell [5]; whom to tell [6]; making a decision, which summarises the previous sections and asks the participant to reflect on responses and make a decision regarding when, what and to whom to disclose. The researcher’s main role will be to record the time taken to read and complete the DA and answer any questions or clarify areas of confusion which arise. If the participant has difficulty in understanding the DA then it will be explained that part of the purpose of the study is to test how easy it is for people to complete the DA and they should use their best guess to answer the questions in it. They will be reminded that there is a chance to discuss any areas which caused confusion at a later stage of the interview. If the participant is not satisfied with this explanation then a choice of what the statement could mean will be given. If this is not sufficient then a more prescriptive set of instructions will be provided, with the researcher guiding the participant through the DA as an interview if necessary. Following this meeting, the participant will be given their DA to keep and continue to receive standard support from the vocational advisers, as per the control condition.

### Control

We have chosen to use a treatment as usual (TAU) control condition, as this provides a fair comparison with routine practice by vocational advisers, and will answer the question of whether use of the DA in addition to current standard practice is superior to standard practice alone. Employment advisers often discuss disclosure needs with those clients seeking competitive employment, so that TAU will in many cases include an unstructured discussion of some of the content covered by the DA. Some vocational advisers using the supported employment model encourage disclosure as part of the process of obtaining work. However, advisers do not routinely provide information about discrimination following disclosure and employees’ rights; instead they do so in response to questions from a client. More broadly, TAU consists of an initial assessment of work history, qualifications, employment and career goals and clinical information. Advisers then assist with augmenting job seeking skills, finding training courses and voluntary placements and providing follow up to those in work. Support for people in work includes helping with requests for workplace adjustments when the need for these arises.

### Objectives

The aims are to determine whether a full scale RCT of the CORAL decision aid tool is justifiable and feasible and to optimise its design [15,16]. Specific objectives are to (i) establish parameters of a future trial, by examining the effect of the DA on decisional conflict regarding disclosure both immediately and at 3 months post first use and determining the optimal primary outcome for a full scale trial from a range of employment related measures; (ii) optimise the evaluation, by testing study procedures, including: the sample that can be recruited and retained using the inclusion and exclusion criteria; the resource implications in terms of full time equivalent needed for research assistants to recruit sufficient numbers and carry out the study procedures in the time available; whether the batch of measures is acceptable vs. leads to too much respondent burden; the use of a questionnaire on employment and disclosure related activities; the randomisation method, in this case individual level randomisation, including the methods for avoiding and measuring contamination; recruitment and retention methods for service users and vocational counsellors; the choice of measures for a full scale trial based on results of qualitative interviews with participants; (iii) optimise the intervention, by collecting feedback on the DA form a larger sample than was used in the previous pilot.

### Hypotheses

**Figure 2:**
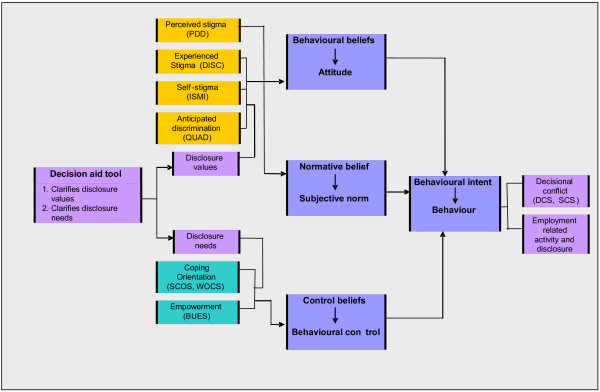
Mechanisms of DA intervention.

While we shall conduct hypothesis testing this is not the main objective of the study, as this is the purpose of the future large scale trial. The reporting of this study will not therefore emphasize the results of hypothesis testing over the reporting of whether the objectives were met [17]. Our hypotheses are derived from the conceptual model in Figure 2 and fall into three groups. Regarding the effect of the intervention, we expect that, regardless of what the disclosure decision is, having a lower level of decisional conflict and being at a later stage of decision will allow the participant to take action regarding seeking work, resulting in a greater level of empowerment and a reduction in behavioural withdrawal. Thus, compared to the control group, those in the intervention group will show at 3 months [1]: lower decisional conflict regarding disclosure [2]; a later stage of decision making regarding disclosure [3]; higher frequencies of work related actions [4]; a higher level of empowerment [5] lower frequencies of withdrawal as a response to stigma (in any context rather than just employment). The model shown in Figure 2 will be investigated using process evaluation and qualitative data collection to identify the most robust associations to carry forward into a final modified and simplified version of the model to be used in the subsequent full scale RCT. 

The DA is not designed either to cause an increase or decrease in rates of disclosure to an actual or potential employer but may have either effect. It may increase disclosure through increasing knowledge about being able to request workplace adjustments. On the other hand, knowing that there is a choice that can be made regarding disclosure may make some people feel more confident about not disclosing. We will therefore study the relationship between use of the DA and subsequent disclosure and use qualitative methods to explore any apparent effect or lack thereof.

### Primary outcome measures

#### Decisional conflict

The decisional conflict scale measures personal perceptions of: uncertainty in choosing between options; modifiable factors contributing to uncertainty including feeling uninformed, lack of clarity about personal values and feeling unsupported in decision making; and effective decision making such as feeling the choice is informed, values-based, likely to be implemented and expressing satisfaction with the choice. The 16 item version of the scale is the most commonly used [18]. A total score and 5 sub-scores (uncertainty; informed; values clarity; support and effective decision) are generated. Scores exceeding 37.5/100 are associated with decisional delay or feeling unsure about implementation.

#### Stage of decision making

The stage of decision making scale measures the individual’s readiness to engage in decision making [19]. It consists of 1 item with 6 response options anchored at 1 (haven’t started to think about the choices) and 6 (have already made a decision and am unlikely to change my mind).

#### Employment related outcomes

We will administer a questionnaire to assess the occurrence and frequency of employment related activity and disclosures made to employers. The baseline interviews will be used to pilot this questionnaire. Its content will cover the previous 3 months regarding: appointments made and kept with the vocational counsellor; applications submitted; interviews attended; jobs offered and accepted; verbal disclosure and its timing e.g. at interview, post offer, after starting employment; written disclosure and its timing e.g. on application forms or occupational health forms; requests for workplace adjustments and the outcome of such requests; job loss and reason for loss; other employment related activity such as training e.g. on CV completion; non-competitive employment and volunteering; and income or job title. We will measure employment rates pre- and post-intervention in each group. We will ask about income from employment; if people are reluctant to disclose this we will assume salary levels based on the job titles and these salary levels will be used to reflect productivity gains. The eight item short version of Work Limitations Questionnaire [20] will also be included for participants to self-assess work performance and these data will be used to make estimates of productivity costs through work limitations whilst at work.

### Secondary outcome measures

#### Empowerment

The Boston University Empowerment Scale (BUES) 17-item version consisting of the self-esteem-self-efficacy and power-powerlessness subscales of the original 28 item scale used in a recent validation study [21].

#### Withdrawal

We shall use the 9 item withdrawal scale of the Stigma Coping Orientation scales [22].

### Predictors of intention to disclose: behaviour beliefs

#### Experienced discrimination

The Discrimination and Stigma Scale [3,13] is a 35 item, interview-based scale. It contains a global scale and 4 subscales: 1) Unfair treatment; 2) Stopping self; 3) Overcoming stigma and 4) Positive treatment. We will use the 22 item Unfair treatment subscale only, which has 21 specific items and one item where other experiences can be recorded.

#### Anticipated discrimination

The Questionnaire on Anticipated Discrimination (QUAD) ( http://www.sapphire.iop.kcl.ac.uk) is a self-complete measure with 14 items which address ‘areas of anticipated discrimination’. It asks participants to provide a rating of whether they expect to be treated unfairly in various areas of life.

#### Self-stigma

The Internalised Stigma of Mental Illness Scale (ISMI) is a 29-item measure that assesses mental health service users’ experience of internalised stigma. It is composed of 5 sub-scales: Alienation, Stereotype Endorsement, Perceived Discrimination, Social Withdrawal and Stigma Resistance [21].

### Normative beliefs

#### Perceived stigma

The Perceived Devaluation and Discrimination Scale (PDD) is a 12-item, uni-dimensional, scale which measures the extent to which a person believes that most people will devalue or discriminate against someone with a mental illness [23,24];.

### Control beliefs

#### Empowerment

The 17 item BUES will be used as above.

#### Coping orientation

Link et al. devised 5 short coping scales with a total of 20 items (the Stigma Coping Orientation Scales) to assess various approaches to coping with the stigma associated with mental illness. Five different coping orientations are assessed: secrecy, withdrawal, educating, challenging and distancing [22,25]. More general coping orientation will be assessed using the revised Ways of Coping Questionnaire (WCQ), a self-report instrument which asks participants to think about a recent stressor and rate the extent to which they used each of 24 behaviours to cope with it [26]. A revised factor structure for the scale, as used with people with a diagnosis of schizophrenia, has been proposed and will be used [27].

### Qualitative data collection

#### Intervention group participants

Interviews will be conducted with up to 15 participants after outcome measures have been administered. This will explore the perceived impact of using the decision aid with respect to (i) actual and intended disclosure; (ii) job seeking behaviour and (iii) other behaviour e.g. disclosure to others. We shall also enquire about experiences over the follow up period which have modified or reinforced intentions regarding disclosure or actual disclosure. Interviews will be recorded and transcribed.

#### Control group participants

Interviews will be conducted with up to 6 consenting participants after outcome measures have been administered. This will explore actual and intended disclosure in relation to employment and others. While this was a focus of previous qualitative work with service users there have been subsequent changes both in relevant legislation (the introduction of the Equality Act 2010) and in the job market, which has contracted. Both of these factors may have influenced disclosure intentions and behaviours.

#### Vocational adviser interviews

Once participant follow up is completed we shall interview a minimum of one adviser from each of the four boroughs. The aim of these will be [1] to better describe treatment as usual with respect to advising service users about disclosure [2]; to identify whether any contamination has occurred, either due to specific use of the DA or due to an increased focus on disclosure as a result of the study. The interview will therefore cover: knowledge of relevant legislation; attitudes about what service users should do with respect to disclosure; and practice with respect to what advice is given, the extent to which the advice is tailored to the client, and whether advice is given routinely versus in response to client requests.

Transcripts of the qualitative interviews will be coded using NVivo and thematic analysis will be used to identify participants’ perceptions of the impact of use of the DA and the impact of subsequent experience on intentions and behaviour regarding disclosure.

### Sample size

The heuristic sample size for pilot studies is 30 per group [16]. However, we have elected to use a larger sample size so that we can test the effect of the DA with respect to a meaningful reduction in decisional conflict. The pre-post difference detected on the Decisional Conflict Scale in a preliminary study [13] was found to be 16.5 points in the intervention group, with a standard deviation of 17.5. Estimating the effect of treatment as usual (i.e. vocational advisor standard support) at 4 points, this gives a standardised effect size of 0.71. Group sizes of 32 at the follow-up are needed to have 80% power to detect a difference of this size or greater at the 5% significance level. Further to test our exploratory hypotheses and refine our conceptual model, increasing from 64 (2×32) to 70 participants at follow up will allow logistic regression analysis to study relationships between baseline predictors and disclosure using 6 variables (plus the group variable), using the minimum of 10 per variable. Since we anticipate some loss to follow up at 3 months we shall aim to recruit 40 per group i.e. a total of 80 participants. Qualitative interview sample sizes are based on our expectations for the number of interviews required to cover a range of experiences and to reach the point where no new themes are identified in the analysis.

### Recruitment and randomisation

#### Ethics approval

The study has been approved by the National Research Ethics Service Committee East of England – Essex.

#### Recruitment

Employment advisers attached to community mental health teams and IAPT services will be asked to inform people on their caseload about the study by giving them a flyer summarising the main points of the study. With service users’ permission the adviser will pass on contact details of interested service users to the study research worker. The worker will contact the service user to determine their eligibility, provide further study information to those eligible, offer a period of time for consideration and document informed consent from those eligible and wishing to enter the study.

#### Sequence generation

Block randomisation with randomly varying block sizes will be used. We shall stratify by [1] referral source i.e. primary vs. secondary mental health services, since these groups are likely to differ with respect to severity of illness, and [2] by length of time out of work (less vs. more than 12 months), as this affects chances of regaining employment.

#### Allocation concealment

The sequence is concealed by the KCL Clinical Trials Unit from the research team.

#### Implementation

All randomisation will be undertaken via the independent Clinical Trials Unit (CTU) (which has been awarded full CTU registration by UKCRC) using an online randomisation system. They will not be provided with identifying information about service users. Participants will be enrolled by research workers blind to allocation while the CTU will inform only the researchers carrying out the intervention of allocation.

### Blinding

The blinded research workers are located in a separate office to those who are non-blind. It is not possible to keep participants blind to the intervention. To ensure the intervention occurs in as close a manner as possible to that in routine practice while minimising contamination, intervention group participants will be allowed to discuss the DA with vocational advisers but will be asked not to give them a copy. This means that vocational advisers cannot be blinded and therefore only the research team will be blind. At follow-up assessment, participants will be asked not to reveal their allocation status, and at the end of the interview the rater will record their guess about the service user’s allocation status. This will allow researcher blindness to be estimated.

### Analysis plan

The principal analysis of effectiveness will compare the primary and secondary outcome measures at 3 months. Changes in Decisional Conflict Scale scores are not prone to cut-off limits and are expected to be normally distributed. For both this and other normally distributed continuous outcome measures, an independent two sample t-test will be used to compare the change in effects (T1-T0) for the two groups (control and intervention). When continuous variables are predicted to be skewed (e.g. number of disclosures made), these can be tested using a two-sample Wilcoxon rank-sum test. The differences in number of disclosures made (T1-T0) for the two groups (control and intervention) can be compared. While items such as job offers and disclosure will be too infrequent to provide reliable comparisons, others such as appointments kept with the vocational adviser and submitted applications will be used as proxy measures for future employment outcomes. We will use confidence intervals for the effect sizes for these employment related activities to judge whether a full scale trial is indicated.

Attendance and dropout rates can be compared in terms of their baseline characteristics to see whether there are any obvious differences between retained and dropout participants. Adjustments to these analyses will be made to compensate for multiple testing. To study factors predicted by our model (see Figure 2) to be associated with disclosure we will use logistic regression. Each instrument will be studied to see whether it performs well enough for inclusion in a future trial. A correlation matrix will be formed from the items to calculate internal consistency and reliability, and to assess the appropriateness of forming composite or summary scores for previously defined sub-scales. Along with the histograms of each subscale, the data allow assessment of the appropriateness (particularly ceiling and floor effects) of the measures. Variables that perform well (and that appear not to be measuring the same thing) will be included in the logistic regression model, while adjusting for gender and diagnosis as these have been found to be associated with disclosure in other studies [28,29].

## Discussion

This study will not include consideration of the cost-effectiveness of the DA for two reasons. First, the methods for data collection for service and societal costs we would use in a full scale trial have been well tested. Second, since the current trial cannot include hypothesis testing we are also unable to determine cost effectiveness. We will however identify any extra resources e.g. employment advisor time required to implement the intervention and attach costs to these. Developmental costs of the intervention could be included but apportioned over a large number of people these would be minimal.

## Competing interests

The authors report no competing interests.

## Authors’ contributions

CH led on trial design and drafting the protocol. EB led on developing the decision aid and conceptual model. SC, FL, RP, OS, CM and MS assisted with trial design. PW advised on the statistical analysis plan. JM led on the qualitative data collection and analysis plan. GT conceived the study. All authors contributed to drafting the protocol and approved the final manuscript.

## Pre-publication history

The pre-publication history for this paper can be accessed here:

http://www.biomedcentral.com/1471-244X/12/133/prepub
